# Independent Associations of Diabetes Stigma and Perceived Social Support with Depression and Anxiety Among Adults with Type 2 Diabetes

**DOI:** 10.3390/healthcare14162486

**Published:** 2026-08-11

**Authors:** Soner Yeşilyurt, Olcay Şenay

**Affiliations:** 1 Department of Internal Medicine, Taksim Training and Research Hospital, University of Health Sciences, Istanbul 34433, Türkiye; 2 Department of Psychiatry, Bakırköy Prof Mazhar Osman Training and Research Hospital for Psychiatry, Neurology, and Neurosurgery, Istanbul 34147, Türkiye; olcaysenay8@gmail.com

**Keywords:** type 2 diabetes mellitus, diabetes stigma, perceived social support, depression, anxiety, insulin therapy

## Abstract

**Background/Objectives:** Depression and anxiety are common among individuals with type 2 diabetes mellitus (T2DM) and are associated with poorer clinical outcomes. Although diabetes stigma and perceived social support have been linked to psychological distress in T2DM, they have largely been investigated separately and with limited adjustment for potential confounders. This study aimed to examine whether diabetes stigma and perceived social support are independently associated with depression and anxiety after adjustment for sociodemographic and clinical confounders and, secondarily, to evaluate the association of insulin use with these psychological outcomes. **Methods:** In this single-center, cross-sectional study, 209 adults with T2DM attending a tertiary internal medicine outpatient clinic were enrolled. Diabetes stigma was assessed using the Type 2 Diabetes Stigma Assessment Scale (DSAS-2), depression using the Beck Depression Inventory (BDI), anxiety using the Beck Anxiety Inventory (BAI), and perceived social support using the Multidimensional Scale of Perceived Social Support (MSPSS). Mann–Whitney U tests, Spearman correlation analyses, and two-step hierarchical linear regression models were performed. **Results:** Diabetes stigma was positively correlated with depression (ρ = 0.351) and anxiety (ρ = 0.432), whereas perceived social support was negatively correlated with both depression (ρ = −0.394) and anxiety (ρ = −0.275) (all *p* < 0.001). After adjustment for sociodemographic and clinical covariates, the addition of diabetes stigma and perceived social support explained an additional 21.4% of the variance in depression (ΔR^2^ = 0.214) and 17.9% of the variance in anxiety (ΔR^2^ = 0.179) (both *p* < 0.001). Both diabetes stigma and perceived social support remained independently associated with depression and anxiety, whereas insulin use was not independently associated with either outcome. **Conclusions:** Diabetes stigma and perceived social support exhibit independent and additive associations with depression and anxiety among individuals with T2DM. Integrating psychosocial assessment, stigma-reduction strategies, and interventions aimed at strengthening social support into routine diabetes care may contribute to improved psychological well-being in this population.

## 1. Introduction

Type 2 diabetes (T2DM) is a chronic, multisystem metabolic disease whose global prevalence is steadily increasing; current estimates project that the global burden of diabetes in adults will continue to rise over the coming decades [1]. T2DM is accompanied not only by metabolic and macro/microvascular complications but also by a substantial psychological burden. Depression is markedly more common in patients with T2DM than in the general population [2], and anxiety disorders and anxiety symptoms are also reported at elevated rates in this patient group [3]. These psychiatric comorbidities, beyond reducing quality of life, adversely affect self-care behaviors, treatment adherence, and glycemic control [4]; a bidirectional relationship between diabetes and mood and anxiety symptoms has been described [5,6]. Therefore, current standards of care recommend psychosocial assessment as an integral part of diabetes follow-up [5].

Among the constructs that seek to explain the psychological burden of T2DM, diabetes stigma is attracting increasing attention. Diabetes stigma encompasses experiences of negative judgements attributed to the disease, as well as blame, exclusion, and devaluation [7], and can be measured with the T2D Stigma Assessment Scale (DSAS-2), a valid T2DM-specific instrument [8]. Evidence from systematic reviews and meta-analyses has shown that diabetes stigma is associated with poorer psychological, clinical, and behavioral outcomes [9], and this adverse effect has also been demonstrated for medication adherence and quality of life [10]. International consensus statements have identified ending diabetes stigma and discrimination as a priority goal [11], and the association between stigma and adverse clinical outcomes has been confirmed across different contexts [12]. Moreover, diabetes stigma is not limited to a single dimension and may overlap with other forms of stigma, such as weight stigma [13].

Another construct that shapes the psychological burden is perceived social support. This concept, assessed with the Multidimensional Scale of Perceived Social Support (MSPSS), encompasses perceived support from family, friends, and a significant other [14]. Low social support has been associated with an increased risk of depression in patients with T2DM; it has been suggested that social support may serve a protective, buffering function [15]. However, stigma and social support have most often been addressed independently of each other, and comparatively few studies have examined their relative and distinct contributions to depression and anxiety within a single model adjusted for sociodemographic and clinical confounders [16,17,18]. Yet these two constructs may overlap conceptually; while the experience of stigma may weaken social relationships and social support networks [19], strong social support may attenuate the effect of stigma. Therefore, evaluating both constructs in the same model, with adjustment for confounders, is necessary to distinguish which is independently associated with these outcomes.

Another contentious issue is the relationship between insulin use and psychological burden. The initiation of insulin therapy has been associated with psychological insulin resistance and stigma, owing to its perception as an indicator of disease progression, injection anxiety, and societal judgements [20,21,22]. It has been reported that stigma levels may be higher in patients with T2DM who use insulin [23], but it is unclear whether these associations arise from insulin itself or from accompanying psychosocial and clinical factors. This uncertainty necessitates further examination of the association between insulin and psychological burden under multivariable control.

Guided by these considerations, the primary objective of this cross-sectional study was to determine whether diabetes stigma and perceived social support are independently associated with depression and anxiety after adjustment for relevant sociodemographic and clinical confounders. The secondary objective was to examine whether insulin-containing treatment is associated with diabetes stigma, depression, anxiety, and perceived social support, and whether these associations remain independent after multivariable adjustment.

## 2. Materials and Methods

### 2.1. Study Design and Participants

This study was designed as a single-centre, cross-sectional, observational study. Participants were enrolled by consecutive sampling between 1 February 2024 and 1 February 2025 from among adult patients with T2DM presenting to the Internal Medicine Outpatient Clinic of Istanbul Taksim Training and Research Hospital. The inclusion criteria were being aged between 18 and 65 years, having a physician-confirmed diagnosis of T2DM for at least 6 months, providing written consent to participate in the study, and having the cognitive capacity to complete the self-report scales in full. Patients with a diagnosis of T1DM or secondary diabetes, those with advanced-stage chronic disease (chronic renal failure, chronic liver disease, or malignancy), those with physician-diagnosed psychiatric disorders identified from medical records and confirmed by self-report during the study interview, those with alcohol or psychoactive substance use, and those with cognitive impairment severe enough to prevent completion of the questionnaires were excluded. In accordance with these criteria, 236 patients were enrolled. Twenty-seven were excluded from the analysis owing to incomplete forms, and the final analyses were conducted on 209 patients. These eligibility criteria were selected to minimize potential confounding from severe systemic illnesses, pre-existing psychiatric disorders, and conditions that could interfere with the reliable completion of the study questionnaires.

The study was conducted in accordance with the principles of the Declaration of Helsinki and was approved by the Clinical Research Ethics Committee of Gaziosmanpaşa Training and Research Hospital (Date: 27 December 2023; Decision No: 188). Written informed consent was obtained from all participants before data collection.

### 2.2. Study Measures

All questionnaires used in this study were internationally established instruments that had previously undergone standardized cross-cultural adaptation and psychometric validation for the Turkish population. These validation studies included forward–backward translation, expert review, assessment of construct validity, and evaluation of internal consistency, demonstrating psychometric properties comparable to those of the original versions. Therefore, the validated Turkish versions were considered appropriate for use in the present study. All questionnaires were administered using their previously validated Turkish versions and were cited and used in accordance with the recommendations of the original developers and the validated Turkish adaptation studies.

The sociodemographic and clinical characteristics of the participants were recorded using a structured information form prepared by the researchers, which included age, sex, marital status, educational level, employment status, income level, smoking status, height (cm), weight (kg), duration of diabetes, family history of diabetes, family history of psychiatric disorders, type of antidiabetic treatment, and self-reported general health perception.

Diabetes stigma was assessed with the DSAS-2, originally developed by Browne et al. [8]. The validated Turkish version demonstrated excellent internal consistency (Cronbach’s α = 0.92) [24]. The scale comprises 19 items, each rated on a 5-point Likert-type scale, and the total score ranges from 19 to 95, with higher scores indicating greater stigma.

Depressive symptoms were measured with the Beck Depression Inventory (BDI) [25]. The validated Turkish version demonstrated satisfactory internal consistency (Cronbach’s α = 0.80) [26]. The total score of the 21-item scale ranges from 0 to 63, and severity is classified as minimal (0–9), mild (10–16), moderate (17–29), and severe (30–63).

Anxiety symptoms were assessed with the Beck Anxiety Inventory (BAI) [27]. The validated Turkish version demonstrated excellent internal consistency (Cronbach’s α = 0.93) [28]. The total score of the 21-item scale ranges from 0 to 63, and severity is defined as minimal (0–7), mild (8–15), moderate (16–25), and severe (26–63).

Perceived social support was measured with the MSPSS [14]. The validated Turkish version demonstrated good internal consistency (Cronbach’s α = 0.89) [29]. The 12-item scale is rated on a 7-point Likert-type scale, and the total score ranges from 12 to 84, with higher scores reflecting greater perceived social support.

### 2.3. Statistical Analysis

Data was analysed using IBM SPSS Statistics 26.0 software. The conformity of continuous variables to a normal distribution was assessed with the Shapiro–Wilk test. Continuous variables that did not show a normal distribution were summarized as the median (first quartile; third quartile [Q1; Q3]), and non-parametric methods were preferred for between-group comparisons. Categorical variables were presented as numbers and percentages (%).

Multi-category variables were dichotomized before analysis: educational level as low versus middle-to-high; income level as low versus middle-to-high; duration of diabetes as <10 years versus ≥10 years; perceived general health as good versus fair-to-poor; antidiabetic treatment as insulin-containing (±oral antidiabetic [OAD]) versus OAD only; and smoking as current smoker versus non-/ex-smoker. This approach was adopted to facilitate interpretation, reduce the number of parameters relative to the sample size, and improve the stability of the multivariable regression models. The selected cut-points were based on clinically meaningful or commonly used categories where applicable (e.g., diabetes duration and treatment status), whereas the remaining variables were collapsed into broader categories to avoid sparse subgroup sizes. The Mann–Whitney U test was used to compare two independent groups. Relationships among continuous variables were examined with the Spearman rank correlation coefficient (ρ).

To evaluate the independent association of stigma and perceived social support with depression and anxiety, two-step hierarchical multiple linear regression analyses were performed with the BDI and Beck Anxiety Inventory (BAI) as dependent variables. In the first step, nine covariates (age, sex, educational level, income level, body mass index (BMI), duration of diabetes, family history of psychiatric disorders, type of treatment, and perceived general health) were entered into the model; in the second step, the DSAS-2 and MSPSS were added. For the regression analyses, diabetes duration was entered as a dichotomous variable (<10 years vs. ≥10 years), consistent with the predefined categorization applied throughout the study. The reference groups for the categorical predictors were defined as female (sex), low (education), low (income), no (family history of psychiatric disorders), OAD only (treatment), and good (general health). The contribution of the variables added in the second step was assessed with the change in R^2^ (ΔR^2^) and the corresponding change in F (ΔF). Multicollinearity in the models was examined using the variance inflation factor (VIF).

To examine the determinants of stigma level, a multiple linear regression model was constructed with the DSAS-2 as the dependent variable and the nine covariates together with the MSPSS as independent variables. In addition, among insulin-treated patients, dose subgroups defined by the number of daily injections (low, 1–2/day; high, ≥3/day) were compared using the Mann–Whitney U test. For all analyses, statistical significance was set at a two-sided *p* < 0.05.

## 3. Results

### 3.1. Sample Characteristics

The 209 patients had a median age of 56 (51; 62) years, and 110 (52.6%) were men. The median BMI was 28.7 (26.0; 31.7) kg/m^2^, and the median duration of diabetes was 8 (3; 12) years. Eighty-one patients (38.8%) were receiving insulin-containing treatment, and 155 (74.2%) rated their general health as fair or poor. Median scale scores were 41 (37; 51) for the DSAS-2, 10 (6; 15) for the BDI, 22 (11; 26) for the BAI, and 68 (57; 80) for the MSPSS. A BDI score of ≥10 was present in 116 patients (55.5%) and a score of ≥17 in 44 (21.1%); for the BAI, 175 patients (83.7%) scored ≥8 and 137 (65.6%) scored ≥16. On the Shapiro–Wilk test, all continuous variables deviated significantly from a normal distribution (*p* < 0.001). Further sample characteristics are presented in Table 1.

### 3.2. Sociodemographic and Clinical Comparisons

BDI scores were higher in women than in men (*p* = 0.011), in those with a low educational level (*p* = 0.025), in the lower income group (*p* = 0.019), in current smokers (*p* = 0.049), in those with a family history of psychiatric disorders (*p* = 0.001), and in those who rated their general health as fair or poor (*p* < 0.001). BAI scores were higher in women (*p* = 0.028), in the lower income group (*p* = 0.003), in those with a family history of psychiatric disorders (*p* = 0.005), in those receiving insulin-containing treatment (*p* = 0.005), and in those who rated their general health as fair or poor (*p* = 0.019). DSAS-2 scores were higher in the lower income group (*p* = 0.015) and in those receiving insulin-containing treatment (*p* = 0.036); the difference according to duration of diabetes did not reach significance (*p* = 0.052). DSAS-2 scores did not differ significantly according to marital status, employment status, or family history of diabetes. MSPSS scores were lower in unmarried participants than in married participants (*p* = 0.002) and were also lower in current smokers (*p* = 0.006), in those with a family history of psychiatric disorders (*p* = 0.033), and in those who rated their general health as fair or poor (*p* = 0.001). All comparisons are presented in Table 2.

### 3.3. Correlations

The DSAS-2 showed a positive correlation with the BDI (ρ = +0.351, *p* < 0.001) and the BAI (ρ = +0.432, *p* < 0.001), and a negative correlation with the MSPSS (ρ = −0.172, *p* = 0.013). A positive correlation was found between the BDI and the BAI (ρ = +0.534, *p* < 0.001). The MSPSS showed a negative correlation with both the BDI (ρ = −0.394, *p* < 0.001) and the BAI (ρ = −0.275, *p* < 0.001). The correlation coefficients are presented in Table 3.

### 3.4. Hierarchical Regression Analyses

*BDI model.* In the first step, the model with nine covariates was significant (R^2^ = 0.176, F(9, 199) = 4.73, *p* < 0.001). In the second step, in which the DSAS-2 and MSPSS were added, the explained variance rose to R^2^ = 0.390 (F(11, 197) = 11.45, *p* < 0.001); this increase was significant (ΔR^2^ = 0.214, ΔF (2, 197) = 34.55, *p* < 0.001). In the full model, the DSAS-2 (B = +0.16, 95% CI +0.10 to +0.22, *p* < 0.001) and the MSPSS (B = −0.16, 95% CI −0.22 to −0.11, *p* < 0.001) were significant predictors. In addition, a family history of psychiatric disorders (B = +3.09, *p* = 0.016), the perception of general health as fair/poor (B = +3.25, *p* = 0.004), and age (B = +0.13, *p* = 0.048) were significant. Insulin-containing treatment was not significant (B = −0.39, *p* = 0.703).

*BAI model.* The first step was significant (R^2^ = 0.146, F(9, 199) = 3.79, *p* < 0.001). In the second step, the explained variance rose to R^2^ = 0.325 (F(11, 197) = 8.62, *p* < 0.001); the increase was significant (ΔR^2^ = 0.179, ΔF (2, 197) = 26.06, *p* < 0.001). In the full model, the DSAS-2 (B = +0.25, 95% CI +0.16 to +0.33, *p* < 0.001), the MSPSS (B = −0.13, 95% CI −0.21 to −0.05, *p* = 0.001), and a family history of psychiatric disorders (B = +4.41, *p* = 0.011) were significant predictors. Insulin-containing treatment was not significant (B = +2.14, *p* = 0.123). In all models, the VIF values were below 1.30 (1.05–1.25). The regression results are presented in Table 4.

### 3.5. Insulin Use and the Study Scales

In patients receiving insulin-containing treatment (n = 81), compared with those receiving OAD only (n = 128), the DSAS-2 score [43.0 (38.0; 57.0) vs. 40.0 (28.0; 50.2), *p* = 0.036] and the BAI score [24.0 (14.0; 30.0) vs. 19.0 (10.0; 24.0), *p* = 0.005] were higher. The BDI score [11.0 (7.0; 16.0) vs. 10.0 (5.0; 15.0), *p* = 0.308] and the MSPSS score [68.0 (57.0; 83.0) vs. 67.0 (58.5; 80.0), *p* = 0.973] did not differ between the two groups. After adjustment for covariates, insulin-containing treatment was not independently associated with the DSAS-2 (B = +3.66, 95% CI −0.85 to +8.17, *p* = 0.111), the BAI (B = +2.14, 95% CI −0.59 to +4.88, *p* = 0.123), or the BDI (B = −0.39, 95% CI −2.40 to +1.62, *p* = 0.703). Among patients using insulin, no difference was detected on any of the four scales between the low-dose (1–2/day, n = 52) and high-dose (≥3/day, n = 29) subgroups defined by the number of daily injections (DSAS-2 *p* = 0.800; BDI *p* = 0.693; BAI *p* = 0.921; MSPSS *p* = 0.740).

### 3.6. Correlates of Stigma

A multiple linear regression model was constructed with the DSAS-2 as the dependent variable and the nine covariates and the MSPSS as predictors; the model was significant (R^2^ = 0.121, F(10, 198) = 2.72, *p* = 0.004). In this model, income level (middle-to-high vs. low; B = −4.99, 95% CI −9.38 to −0.60, *p* = 0.026) and the MSPSS (B = −0.18, 95% CI −0.31 to −0.05, *p* = 0.007) were significant predictors.

## 4. Discussion

In this cross-sectional study, the relationship of diabetes stigma and perceived social support with depression and anxiety was examined in patients with T2DM, with adjustment for a broad set of sociodemographic and clinical confounders within the same model. The main finding is that both stigma and perceived social support remained independently associated with both depression and anxiety even after adjustment for the nine covariates. Stigma was positively associated with both outcomes, whereas perceived social support was negatively associated, and the additive contribution of the two constructs to the variance in the BDI and BAI was substantial (ΔR^2^ = 0.214 and ΔR^2^ = 0.179, respectively). Going beyond previous studies that demonstrated the relationship of stigma and social support with depression and anxiety separately, this result shows that the two constructs contribute within the same model independently of each other and of traditional confounders. Meta-analytic evidence demonstrating the relationship of diabetes stigma with adverse psychological outcomes [9] and studies reporting that low social support increases the risk of depression in T2DM [15] have largely focused on a single psychosocial construct; our findings, by contrast, show that the variance explained by these two constructs does not overlap and that they therefore complement rather than exclude each other. The low correlation between stigma and perceived social support (ρ = −0.172) indicates that these two constructs represent largely non-overlapping, distinct psychosocial dimensions: stigma reflects a subjective experience of the negative judgement and devaluation attributed to the disease [7], whereas perceived social support represents the relational resources that the individual derives from their environment. This conceptual distinction explains the possibility that social support may be preserved in a patient with high stigma (or vice versa) and helps explain why the two constructs contribute independently to depression and anxiety. These findings align with network analysis results demonstrating depression, anxiety, and diabetes distress form interconnected yet distinct psychosocial constructs [30], and with research underscoring the bidirectional relationship between diabetes and psychological symptoms [6]. The demonstration of the independent contributions of stigma and perceived social support within the same model complements studies suggesting that social support may, to some extent, buffer the effects of stigma [17] and research showing that psychosocial factors can modify the psychological effect of stigma [31].

Several mechanisms have been proposed to explain the association between diabetes stigma and psychological outcomes, although these mechanisms were not directly evaluated in the present study. The internalization of negative judgements about the disease may foster negative self-evaluation and hopelessness; perceived stigma may lead to social withdrawal, concealment of diabetes, and avoidance of self-management behaviors. It has been reported that stigma increases distress by weakening diabetes self-efficacy [16], and stigma also operates as part of a broader psychosocial symptom network in which depression and anxiety are interwoven [30]. Perceived social support has consistently been associated with lower psychological distress and may represent an important psychosocial correlate of depression and anxiety in individuals with T2DM. Previous studies have suggested that social support may buffer the psychological impact of diabetes-related stressors by influencing stress appraisal, strengthening coping resources, and attenuating the association between stigma and quality of life [17,31]. Likewise, low perceived social support has been associated with greater depressive symptoms through potential mediating psychosocial mechanisms [18]. These findings are also consistent with the stress-buffering framework of social support, according to which social support may mitigate the adverse psychological consequences of stressful or discriminatory experiences. In individuals with T2DM, diabetes-related stigma, chronic disease burden, and treatment demands may function as persistent psychosocial stressors, whereas social support may help individuals cope with these challenges by providing emotional, informational, and practical resources. Consistent with this perspective, Ekpor et al. [32] reported that social support moderated the association between healthcare discrimination and quality of life among individuals with T2DM, providing further evidence for the relevance of social support in diabetes-related psychosocial outcomes. Similarly, in the present study, perceived social support remained independently associated with lower depression and anxiety scores after adjustment for diabetes stigma and a broad range of sociodemographic and clinical covariates. However, given the cross-sectional design, these findings should be interpreted as associations rather than evidence of a causal or protective effect, and prospective longitudinal studies are needed to clarify the direction of these relationships.

### 4.1. Insulin Use and Psychological Burden

In bivariate comparisons, patients receiving insulin-containing treatment had higher stigma (*p* = 0.036) and higher anxiety (*p* = 0.005) scores than those receiving OAD only; by contrast, the groups did not differ in terms of depression (*p* = 0.308) or perceived social support (*p* = 0.973). At first glance, this pattern appears consistent with previous reports showing that insulin therapy is associated with psychological burden [23]. However, after adjustment for sociodemographic and clinical confounders, insulin use was not independently associated with stigma (*p* = 0.111), anxiety (*p* = 0.123), or depression (*p* = 0.703). This finding suggests that the higher stigma and anxiety observed among insulin-treated patients in the unadjusted analyses were no longer evident after adjustment for the measured covariates. However, given the cross-sectional design and the potential for residual confounding, these findings should not be interpreted as evidence that insulin treatment itself has no psychological impact.

The lack of any difference in perceived social support indicates that the elevated stigma and anxiety in insulin users cannot be attributed to lower perceived social support but appear instead to be related to factors such as income, family history of psychiatric disorders, and stigma. The absence of a difference on any scale according to dose intensity (1–2 versus ≥3 injections per day) further supports the view that this psychological burden does not increase linearly with the ‘amount’ of insulin. These observations are consistent with the literature emphasizing that psychological insulin resistance and barriers to insulin initiation are largely shaped by perceptions, beliefs, and societal judgements [20,21], and with studies showing that, in patients with T2DM treated with insulin, stigma is associated with negative treatment appraisals [22].

### 4.2. Interpretation of the Sociodemographic and Clinical Findings

In bivariate comparisons, patients in the lower income group had higher depression, anxiety, and stigma scores. In the multivariable models, while income was no longer independently associated with depression or anxiety, it remained independently associated with the stigma level. This pattern suggests that the association of low income with depression and anxiety may be partly explained by intermediate factors such as stigma; indeed, it has been proposed that socioeconomic disadvantage may mediate the psychological burden in T2DM through pathways [32,33]. A family history of psychiatric disorders, in turn, emerged as the most consistent independent covariate for both depression and anxiety [33,34]; this finding reflects the fact that familial/genetic predisposition and shared environmental factors are also associated with psychological burden in the context of diabetes.

Beyond this mediating role, the stigma level itself proved to be socioeconomically structured: low perceived social support was independently associated with stigma alongside low income (overall R^2^ = 0.121, *p* = 0.004), consistent with other reports linking socioeconomic and psychosocial factors to stigma [34,35]. This result shows that diabetes stigma is not only an individual experience but also a reflection of social inequality. Socioeconomic disadvantage may entail both greater exposure to stigma and fewer protective resources (education, health literacy, supportive networks) against this experience. Within this framework, stigma may function as a channel through which socioeconomic inequality is internalized at the individual level: while inequality is ‘translated’ into psychological burden through stigma, low-income patients are at the same time deprived of the support that would alleviate this burden. The greater susceptibility of low-income and inadequately supported patients to stigma, when considered together with studies reporting a high burden of depressive symptoms among individuals with diabetes in developing countries [35,36], indicates that psychosocial interventions should consider not only the individual but also the socioeconomic context.

### 4.3. Clinical Implications

The findings support the importance of routine screening for depression and anxiety in T2DM care; such screening may be prioritized particularly in patients in the lower income group, those with a family history of psychiatric disorders, and those who perceive their general health as poor. Current standards of care already recommend psychosocial assessment as an integral part of diabetes follow-up [5]. The observed independent associations between diabetes stigma and psychological outcomes suggest that these psychosocial factors may be considered during routine diabetes care. However, whether interventions targeting stigma led to improved psychological outcomes remains to be established in prospective interventional studies [11]. The independent association between perceived social support and lower levels of depression and anxiety, together with recent evidence demonstrating that social support moderates the adverse effects of healthcare discrimination on quality of life in individuals with T2DM [32], supports the consideration of assessing patients’ social support resources as part of routine psychosocial evaluation [15]. Furthermore, previous interventional studies have reported that cognitive behavioral therapy-based interventions were associated with improvements in diabetes distress, depression, anxiety, and quality of life [37]. Finally, the absence of an independent association between insulin-containing treatment and psychological outcomes in the adjusted analyses suggests that the observed psychological burden among insulin-treated patients may be influenced by broader clinical and psychosocial factors. This finding should be interpreted cautiously as residual confounding, limited statistical power, and the absence of detailed measures of diabetes severity, glycemic control, diabetes-related complications, treatment adherence, and history of hypoglycemia may have influenced the observed associations. Therefore, these findings should not be interpreted as evidence that insulin-containing treatment has no psychological relevance.

### 4.4. Limitations

This study has several limitations. First, the cross-sectional design precludes causal inference; therefore, the direction of the associations between diabetes stigma, perceived social support, depression, and anxiety cannot be determined. Second, this was a single-center study conducted in a tertiary outpatient clinic, which may limit the generalizability of the findings. Third, participants with physician-diagnosed psychiatric disorders were excluded to minimize potential confounding; however, this may have introduced selection bias, potentially underestimating the prevalence and severity of depressive and anxiety symptoms in the study population. Fourth, all psychological variables were assessed using self-report questionnaires and are therefore susceptible to response bias. Fifth, although the analyses adjusted for several sociodemographic and clinical variables, residual confounding cannot be excluded. Important clinical factors, including glycemic control (HbA1c), diabetes-related complications, treatment adherence, history of hypoglycemia, overall diabetes severity, treatment complexity, and comorbidity burden, were not available for inclusion in the models and may have influenced the observed associations. Sixth, the analyses involving insulin-containing treatment and the regression model for diabetes stigma were exploratory and should therefore be interpreted cautiously. In addition, dichotomization of several variables, although performed to improve model stability and facilitate interpretation, may have reduced statistical power and obscured potential dose–response relationships. Finally, although hierarchical linear regression was considered appropriate for the study objectives, additional regression diagnostics and sensitivity analyses were not performed. Therefore, the robustness and generalizability of these findings should be confirmed in larger prospective multicenter studies using more comprehensive clinical data and alternative analytical approaches where appropriate. Future studies with larger sample sizes should evaluate these variables using more detailed categorical or continuous modelling strategies to determine whether additional clinically relevant information can be obtained.

## 5. Conclusions

This study demonstrated that diabetes stigma and perceived social support were independently associated with depression and anxiety among adults with T2DM after adjustment for a broad range of sociodemographic and clinical covariates. The two psychosocial constructs provided complementary information and explained a substantial proportion of the variability in psychological outcomes, whereas insulin-containing treatment was not independently associated with depression or anxiety in the adjusted analyses. However, this finding should be interpreted cautiously because residual confounding, limited statistical power, and unmeasured indicators of disease severity may have influenced the observed associations. Prospective longitudinal studies incorporating more detailed clinical measures are needed to further clarify the relationship between insulin-containing treatment and psychological outcomes. These findings support the inclusion of psychosocial factors in the comprehensive assessment of individuals with T2DM, alongside conventional clinical evaluation. Routine assessment of diabetes stigma and perceived social support may assist in identifying individuals with greater psychological distress who could benefit from further psychosocial evaluation. Because diabetes stigma and perceived social support are potentially modifiable psychosocial factors, they may represent relevant targets for future intervention studies in addition to conventional diabetes management. Although our findings were obtained from a single tertiary-care center, they may have relevance to other healthcare settings; however, the generalizability of these findings requires confirmation in more diverse populations. Importantly, the cross-sectional design precludes conclusions regarding causality. Therefore, prospective longitudinal and interventional studies are needed to determine whether modifying diabetes stigma and perceived social support results in improved psychological and clinical outcomes.

## Figures and Tables

**Table 1:** Sample characteristics (N = 209).

Variable	Value
*Sociodemographic*	
Age (years), median (Q1; Q3)	56 (51; 62)
Sex, n (%)	Male 110 (52.6)/Female 99 (47.4)
Married, n (%)	176 (84.2)
Low educational level, n (%)	106 (50.7)
Employed, n (%)	70 (33.5)
Low-income group, n (%)	111 (53.1)
*Clinical*	
BMI (kg/m^2^), median (Q1; Q3)	28.7 (26.0; 31.7)
Diabetes duration (years), median (Q1; Q3)	8 (3; 12)
Diabetes duration ≥ 10 years, n (%)	96 (45.9)
Family history of diabetes, n (%)	147 (70.3)
Family history of psychiatric disorders, n (%)	36 (17.2)
Insulin-containing treatment, n (%)	81 (38.8)
General health fair/poor, n (%)	155 (74.2)
Current smoker, n (%)	73 (34.9)
*Scales*	
DSAS-2, median (Q1; Q3)	41 (37; 51)
BDI, median (Q1; Q3)	10 (6; 15)
BDI ≥ 10, n (%)	116 (55.5)
BDI ≥ 17, n (%)	44 (21.1)
BAI, median (Q1; Q3)	22 (11; 26)
BAI ≥ 8, n (%)	175 (83.7)
BAI ≥ 16, n (%)	137 (65.6)
MSPSS, median (Q1; Q3)	68 (57; 80)

Q1, first quartile; Q3, third quartile; BMI, body mass index; DSAS-2, Type 2 Diabetes Stigma Assessment Scale; BDI, Beck Depression Inventory; BAI, Beck Anxiety Inventory; MSPSS, Multidimensional Scale of Perceived Social Support.

**Table 2:** Comparison of scale scores by sociodemographic and clinical characteristics.

Characteristic	DSAS-2	BDI	BAI	MSPSS
Sex				
Female (n = 99)	41.0 (38.0; 52.0)	12.0 (7.0; 16.0)	22.0 (14.5; 27.5)	69.0 (58.5; 80.5)
Male (n = 110)	41.0 (35.2; 48.8)	9.0 (5.0; 14.0)	19.0 (8.2; 25.0)	66.0 (57.0; 80.0)
*p*	0.507	0.011 *	0.028 *	0.309
Marital status				
Married (n = 176)	41.0 (38.0; 52.0)	10.0 (5.0; 15.0)	21.5 (10.0; 26.0)	68.0 (59.0; 83.0)
Not married (n = 33)	38.0 (30.0; 45.0)	11.0 (8.0; 18.0)	22.0 (13.0; 28.0)	60.0 (38.0; 69.0)
*p*	0.166	0.075	0.316	0.002 *
Education				
Low (n = 106)	41.0 (38.0; 52.8)	11.5 (7.0; 16.0)	22.0 (13.2; 27.0)	66.0 (57.2; 78.8)
Middle-to-high (n = 103)	41.0 (35.0; 50.0)	9.0 (5.0; 15.0)	21.0 (10.0; 25.5)	68.0 (57.5; 81.5)
*p*	0.390	0.025 *	0.350	0.738
Employment				
Employed (n = 70)	41.5 (38.0; 51.8)	9.5 (5.0; 14.0)	21.0 (8.0; 24.8)	66.5 (55.8; 80.0)
Not working/retired (n = 139)	41.0 (36.5; 51.0)	11.0 (6.0; 16.0)	22.0 (12.5; 27.0)	68.0 (57.5; 80.0)
*p*	0.904	0.050	0.169	0.759
Income				
Low (n = 111)	42.0 (38.0; 57.0)	12.0 (6.0; 17.0)	23.0 (15.0; 27.5)	68.0 (57.5; 80.0)
Middle-to-high (n = 98)	39.5 (28.8; 47.0)	9.0 (5.0; 14.8)	18.0 (8.2; 24.0)	68.0 (57.2; 80.0)
*p*	0.015 *	0.019 *	0.003 *	0.831
Smoking				
Current (n = 73)	41.0 (36.0; 52.0)	11.0 (7.0; 18.0)	21.0 (12.0; 25.0)	64.0 (48.0; 72.0)
Non-/ex-smoker (n = 136)	41.0 (38.0; 51.0)	10.0 (5.0; 15.0)	22.0 (11.0; 27.0)	71.0 (59.8; 83.2)
*p*	0.820	0.049 *	0.984	0.006 *
Diabetes duration				
<10 years (n = 113)	38.0 (35.0; 48.0)	10.0 (6.0; 16.0)	20.0 (11.0; 25.0)	67.0 (57.0; 79.0)
≥10 years (n = 96)	43.0 (38.0; 55.0)	10.0 (6.0; 15.0)	22.0 (11.0; 28.0)	68.5 (57.8; 83.0)
*p*	0.052	0.784	0.270	0.595
Family history of diabetes				
Yes (n = 147)	40.0 (36.0; 50.5)	10.0 (5.5; 15.0)	21.0 (11.0; 26.0)	68.0 (57.0; 80.0)
No (n = 62)	42.0 (38.0; 56.8)	10.5 (6.0; 16.0)	22.0 (10.5; 27.0)	68.0 (59.2; 81.0)
*p*	0.252	0.309	0.733	0.839
Family history of psychiatric disorders				
Yes (n = 36)	42.0 (35.0; 64.5)	14.0 (9.5; 22.0)	25.0 (17.8; 31.2)	61.5 (47.8; 72.5)
No (n = 173)	40.0 (38.0; 50.0)	10.0 (5.0; 15.0)	20.0 (10.0; 25.0)	68.0 (59.0; 82.0)
*p*	0.264	0.001 *	0.005 *	0.033 *
Treatment				
Insulin ± OAD (n = 81)	43.0 (38.0; 57.0)	11.0 (7.0; 16.0)	24.0 (14.0; 30.0)	68.0 (57.0; 83.0)
OAD only (n = 128)	40.0 (28.0; 50.2)	10.0 (5.0; 15.0)	19.0 (10.0; 24.0)	67.0 (58.5; 80.0)
*p*	0.036 *	0.308	0.005 *	0.973
General health				
Good (n = 54)	41.5 (35.8; 48.0)	7.5 (4.0; 10.8)	19.0 (8.2; 24.0)	75.0 (62.0; 84.0)
Fair-poor (n = 155)	41.0 (37.5; 52.0)	11.0 (7.0; 17.5)	22.0 (12.0; 27.0)	66.0 (53.5; 76.5)
*p*	0.903	<0.001 *	0.019 *	0.001 *

Values are median (Q1; Q3). *p* from the Mann–Whitney U test. * *p* < 0.05. DSAS-2, Type 2 Diabetes Stigma Assessment Scale; BDI, Beck Depression Inventory; BAI, Beck Anxiety Inventory; MSPSS, Multidimensional Scale of Perceived Social Support; OAD, oral antidiabetic; Q1, first quartile; Q3, third quartile.

**Table 3:** Correlations among the scales.

	DSAS-2	BDI	BAI	MSPSS
DSAS-2	—			
BDI	+0.351 ***	—		
BAI	+0.432 ***	+0.534 ***	—	
MSPSS	−0.172 *	−0.394 ***	−0.275 ***	—

Values are Spearman’s rank correlation coefficients (ρ). * *p* < 0.05; *** *p* < 0.001. DSAS-2, Type 2 Diabetes Stigma Assessment Scale; BDI, Beck Depression Inventory; BAI, Beck Anxiety Inventory; MSPSS, Multidimensional Scale of Perceived Social Support.

**Table 4:** Hierarchical multiple linear regression for depression (BDI) and anxiety (BAI).

Variable	BDI		BAI	
	B (95% CI)	*p*	B (95% CI)	*p*
Age	+0.13 (+0.00 to +0.26)	0.048 *	+0.03 (−0.15 to +0.21)	0.765
Sex (male vs. female)	−1.38 (−3.26 to +0.51)	0.151	−1.40 (−3.96 to +1.17)	0.283
Education (middle-to-high vs. low)	−1.09 (−3.04 to +0.86)	0.272	+0.57 (−2.09 to +3.22)	0.675
Income (middle-to-high vs. low)	−1.36 (−3.33 to +0.61)	0.174	−1.98 (−4.65 to +0.70)	0.148
BMI	+0.05 (−0.13 to +0.24)	0.564	+0.05 (−0.21 to +0.30)	0.707
Diabetes duration	−0.15 (−0.32 to +0.01)	0.073	+0.08 (−0.15 to +0.31)	0.500
Family history of psychiatric disorders (yes vs. no)	+3.09 (+0.59 to +5.59)	0.016 *	+4.41 (+1.01 to +7.81)	0.011 *
Treatment (insulin vs. OAD)	−0.39 (−2.40 to +1.62)	0.703	+2.14 (−0.59 to +4.88)	0.123
General health (fair-poor vs. good)	+3.25 (+1.06 to +5.44)	0.004 *	+2.31 (−0.67 to +5.28)	0.128
DSAS-2	+0.16 (+0.10 to +0.22)	<0.001 *	+0.25 (+0.16 to +0.33)	<0.001 *
MSPSS	−0.16 (−0.22 to −0.11)	<0.001 *	−0.13 (−0.21 to −0.05)	0.001 *
Step 1	R^2^ = 0.176; F(9, 199) = 4.73; *p* < 0.001		R^2^ = 0.146; F(9, 199) = 3.79; *p* < 0.001	
Step 2	R^2^ = 0.390; F(11, 197) = 11.45; *p* < 0.001		R^2^ = 0.325; F(11, 197) = 8.62; *p* < 0.001	
Change	ΔR^2^ = 0.214; ΔF(2, 197) = 34.55; *p* < 0.001		ΔR^2^ = 0.179; ΔF(2, 197) = 26.06; *p* < 0.001	

B, unstandardized regression coefficient; 95% CI, 95% confidence interval; DSAS-2, Type 2 Diabetes Stigma Assessment Scale; BDI, Beck Depression Inventory; BAI, Beck Anxiety Inventory; MSPSS, Multidimensional Scale of Perceived Social Support; OAD, oral antidiabetic. Reference categories: female (sex), low (education), low (income), no (family history of psychiatric disorders), OAD only (treatment), good (general health). * *p* < 0.05.

## Data Availability

The original contributions presented in this study are included in the article. Further inquiries can be directed to the corresponding author.

## References

[1] Genitsaridi I., Salpea P., Salim A., Sajjadi S.F., Tomic D., James S., Thirunavukkarasu S., Issaka A., Chen L., Basit A. (2026). 11th edition of the IDF Diabetes Atlas: Global, regional, and national diabetes prevalence estimates for 2024 and projections for 2050. Lancet Diabetes Endocrinol..

[2] Wang F., Wang S., Zong Q.Q., Zhang Q., Ng C.H., Ungvari G.S., Xiang Y.T. (2019). Prevalence of comorbid major depressive disorder in Type 2 diabetes: A meta-analysis of comparative and epidemiological studies. Diabet. Med..

[3] Smith K.J., Béland M., Clyde M., Gariépy G., Pagé V., Badawi G., Rabasa-Lhoret R., Schmitz N. (2013). Association of diabetes with anxiety: A systematic review and meta-analysis. J. Psychosom. Res..

[4] Alwhaibi M. (2024). Depression, anxiety, and health-related quality of life in adults with type 2 diabetes. J. Clin. Med..

[5] American Diabetes Association Professional Practice Committee (2025). 5. Facilitating positive health behaviors and well-being to improve health outcomes: Standards of Care in Diabetes—2025. Diabetes Care.

[6] Xu H., Chen Q. (2025). The bidirectional influence between type 2 diabetes mellitus and the state of depression and anxiety. J. Affect. Disord..

[7] Browne J.L., Ventura A., Mosely K., Speight J. (2013). ‘I call it the blame and shame disease’: A qualitative study about perceptions of social stigma surrounding type 2 diabetes. BMJ Open.

[8] Browne J.L., Ventura A.D., Mosely K., Speight J. (2016). Measuring the stigma surrounding type 2 diabetes: Development and validation of the Type 2 Diabetes Stigma Assessment Scale (DSAS-2). Diabetes Care.

[9] Akyirem S., Ekpor E., Namumbejja Abwoye D., Batten J., Nelson L.E. (2023). Type 2 diabetes stigma and its association with clinical, psychological, and behavioral outcomes: A systematic review and meta-analysis. Diabetes Res. Clin. Pract..

[10] Li X., Wu L., Yun J., Sun Q. (2023). The status of stigma in patients with type 2 diabetes mellitus and its association with medication adherence and quality of life in China: A cross-sectional study. Medicine.

[11] Speight J., Holmes-Truscott E., Garza M., Scibilia R., Wagner S., Kato A., Pedrero V., Deschênes S., Guzman S.J., Joiner K.L. (2024). Bringing an end to diabetes stigma and discrimination: An international consensus statement on evidence and recommendations. Lancet Diabetes Endocrinol..

[12] Eitel K.B., Pihoker C., Barrett C.E., Roberts A.J. (2024). Diabetes stigma and clinical outcomes: An international review. J. Endocr. Soc..

[13] Himmelstein M.S., Puhl R.M. (2021). At multiple fronts: Diabetes stigma and weight stigma in adults with type 2 diabetes. Diabet. Med..

[14] Zimet G.D., Dahlem N.W., Zimet S.G., Farley G.K. (1988). The Multidimensional Scale of Perceived Social Support. J. Pers. Assess..

[15] Azmiardi A., Murti B., Febrinasari R.P., Tamtomo D.G. (2022). Low social support and risk for depression in people with type 2 diabetes mellitus: A systematic review and meta-analysis. J. Prev. Med. Public Health.

[16] Xing S., Liu Y., Zhang H., Li B., Jiang X. (2023). The mediating role of diabetes stigma and self-efficacy in relieving diabetes distress among patients with type 2 diabetes mellitus: A multicenter cross-sectional study. Front. Psychol..

[17] Onu D.U., Obi-keguna C.N., Oguguam O.N., Ajaero C.K., Igwe E.J. (2025). Social support may buffer, to an extent, the impact of stigma on health-related quality of life among type 2 diabetes mellitus patients. Discov. Public Health.

[18] Mersha A.B., Shimekaw A., Gebremedhin E., Sewalem J., Bayih A., Demeke A.D., Gebre S., Baradaran H.R. (2025). Diabetes-related distress and associated factors among adult diabetes mellitus patients attending public hospitals in Gedio zone, southern Ethiopia: Mediation analysis. PLoS ONE.

[19] Kato A., Fujimaki Y., Fujimori S., Isogawa A., Onishi Y., Suzuki R., Yamauchi T., Ueki K., Kadowaki T., Hashimoto H. (2017). Psychological and behavioural patterns of stigma among patients with type 2 diabetes: A cross-sectional study. BMJ Open.

[20] Galdón Sanz-Pastor A., Justel Enríquez A., Sánchez Bao A., Ampudia-Blasco F.J. (2024). Current barriers to initiating insulin therapy in individuals with type 2 diabetes. Front. Endocrinol..

[21] Jiang H., Wang Z., Liu K., Yang D., Meng M., Li X., Hao Y. (2025). Influencing factors of psychological insulin resistance among patients with type 2 diabetes: A systematic review and meta-analysis. J. Diabetes Metab. Disord..

[22] Holmes-Truscott E., Browne J.L., Ventura A.D., Pouwer F., Speight J. (2018). Diabetes stigma is associated with negative treatment appraisals among adults with insulin-treated type 2 diabetes: Results from the second Diabetes MILES–Australia (MILES-2) survey. Diabet. Med..

[23] Aslan E.Ö., Toygar İ., Feyizoğlu G., Polat S., Eti Aslan F. (2023). Relationship between the insulin use and stigma in type 2 diabetes mellitus. Prim. Care Diabetes.

[24] İnkaya B., Karadağ E. (2021). Turkish validity and reliability study of type 2 diabetes stigma assessment scale. Turk. J. Med. Sci..

[25] Beck A.T., Ward C.H., Mendelson M., Mock J., Erbaugh J. (1961). An inventory for measuring depression. Arch. Gen. Psychiatry.

[26] Hisli N. (1989). Beck Depresyon Envanteri’nin üniversite öğrencileri için geçerliği, güvenirliği. Psikol. Derg..

[27] Beck A.T., Epstein N., Brown G., Steer R.A. (1988). An inventory for measuring clinical anxiety: Psychometric properties. J. Consult. Clin. Psychol..

[28] Ulusoy M., Şahin N.H., Erkmen H. (1998). Turkish version of the Beck Anxiety Inventory: Psychometric properties. J. Cogn. Psychother..

[29] Eker D., Arkar H., Yaldız H. (2001). Çok Boyutlu Algılanan Sosyal Destek Ölçeği’nin gözden geçirilmiş formunun faktör yapısı, geçerlik ve güvenirliği. Türk. Psikiyatr. Derg..

[30] Zu W., Li F., Ma X., Zhang S., Nie W., Wang L. (2024). Exploring the interconnectedness of depression, anxiety, diabetes distress, and related psychosocial factors in adults with type 2 diabetes: A network analysis. J. Context. Behav. Sci..

[31] Holmes-Truscott E., Ventura A.D., Thuraisingam S., Pouwer F., Speight J. (2020). Psychosocial moderators of the impact of diabetes stigma: Results from the second Diabetes MILES—Australia (MILES-2) study. Diabetes Care.

[32] Ekpor E., Adawudu E.A., Agyen S.A., Romadlon D.S., Akyirem S. (2025). The Moderating Effect of Social Support on the Association Between Healthcare Discrimination and Quality of Life in Persons with Type 2 Diabetes. Healthcare.

[33] Park S.H., Lee Y.B., Lee K.N., Kim B., Cho S.H., Kwon S.Y., Park J., Kim G., Jin S.M., Hur K.Y. (2024). Risk of depression according to cumulative exposure to a low-household income status in individuals with type 2 diabetes mellitus: A nationwide population-based study. Diabetes Metab. J..

[34] Albai O., Timar B., Braha A., Timar R. (2024). Predictive factors of anxiety and depression in patients with type 2 diabetes mellitus. J. Clin. Med..

[35] Taher T.M.J., Ahmed H.A., Abutiheen A.A., Alfadhul S.A., Ghazi H.F. (2023). Stigma perception and determinants among patients with type 2 diabetes mellitus in Iraq. J. Egypt. Public Health Assoc..

[36] Aschner P., Gagliardino J.J., Ilkova H., Lavalle F., Ramachandran A., Mbanya J.C., Shestakova M., Bourhis Y., Chantelot J.M., Chan J.C.N. (2021). High prevalence of depressive symptoms in patients with type 1 and type 2 diabetes in developing countries: Results from the International Diabetes Management Practices Study (IDMPS). Diabetes Care.

[37] Abbas Q., Latif S., Ayaz Habib H., Shahzad S., Sarwar U., Shahzadi M., Ramzan Z., Washdev W. (2023). Cognitive behavior therapy for diabetes distress, depression, health anxiety, quality of life and treatment adherence among patients with type-II diabetes mellitus: A randomized control trial. BMC Psychiatry.

